# Book Reviews

**Published:** 1799-05

**Authors:** 


POPULAR OR DOMESTIC MEDICINE.

*Medical Admonitions addressed to Families, respecting the Practice of Domestic Medicine, and the Preservation of Health, &c.* by JAMES PARKINSON. 2 vols. together 500 pp. 8vo. 9s. *London.* Dilly.

We have perused the above book with uncommon satisfaction and regret. Satisfaction with the design and subject of the work, as well as the manner in which it is executed: regret that it was not published many years ago. There are, perhaps, very few so sanguine as to expect that quackery can be prevented; though all are convinced that it destroys more in this island than the sword, famine, and pestilence united. To diminish the number of its victims, is the object of the work before us. The author gives the following account of his design, in the beginning of the first volume. "My dear friend, I comply, with the utmost willingness, with your request, to supply you with such information as may prevent you, on the one hand, from unnecessarily incurring the expence of medical attendance in the various trifling ails to which you and your family may be subjected; and on the other, from sacrificing a friend, or perhaps a beloved child, by delay or improper interference, in some insidious disease."

(To be continued in our next Number.)

*Traité de l'education corporelle des Enfans, &c. A Treatise on the Physical Education of Children; or Practical Reflections on the means of obtaining a Bodily Constitution, most suitable to Republicans:* By J. C. DESSESSARTZ, Physician, Member of the National Institute, &c. The second edition enlarged with a Preface and Supplement. viith year. 8vo. pp. 514, (5 livres sewed) *Paris.* Barrois.

Although the first edition of this work was published no less than thirty-three years ago, (1766,) it has not become so extensively known as it well deserves. The supplement, which was not in the former edition, contains excellent precepts for mothers who wish to suckle their own children; a curious and well selected catalogue of books on physical education; and lastly, what no book ought to want, a good index.

*Essai sur les maladies physiques & morales des femmes: An Essay on the Physical and Moral Diseases of Women.*—By B. LAFFECTEUR. 8vo. (1 liv.) *Paris.*

The author in this small volume, endeavours to include all the usual maladies incident to females, in the different ranks and periods of life, as well as the political evils to which the sex are subject in society. Upon the whole, the treatise before us possesses considerable merit; for, besides explaining the nature of the most common female disorders, Cit. Laffecteur ably comments upon the influence of the passions over the physical constitution of women, and forcibly inculcates the necessity of regulating the former for the benefit of the latter. He justly observes, that no one comes in a state of disease from the hands of nature; and that all disorders of the human body are more or less the consequences of the infraction of her laws; hence he draws the necessary inference, that the principal attention ought to be paid to air, climate, clothing, food, and other particulars of diet and regimen.

